# Cancer Epigenetic Biomarkers in Liquid Biopsy for High Incidence Malignancies

**DOI:** 10.3390/cancers13123016

**Published:** 2021-06-16

**Authors:** Cora Palanca-Ballester, Aitor Rodriguez-Casanova, Susana Torres, Silvia Calabuig-Fariñas, Francisco Exposito, Diego Serrano, Esther Redin, Karmele Valencia, Eloisa Jantus-Lewintre, Angel Diaz-Lagares, Luis Montuenga, Juan Sandoval, Alfonso Calvo

**Affiliations:** 1 Biomarkers and Precision Medicine (UBMP) and Epigenomics Unit, IIS, La Fe, 46026 Valencia, Spain; cora_palanca@iislafe.es; 2 Cancer Epigenomics, Translational Medical Oncology (Oncomet), Health Research Institute of Santiago (IDIS), University Clinical Hospital of Santiago (CHUS/SERGAS), 15706 Santiago de Compostela, Spain; aitorrodriguez@me.com (A.R.-C.); angel.diaz.lagares@sergas.es (A.D.-L.); 3 Roche-CHUS Joint Unit, Translational Medical Oncology Group (Oncomet), Health Research Institute of Santiago (IDIS), 15706 Santiago de Compostela, Spain; 4 CIBERONC, ISCIII, 28029 Madrid, Spain; susana.torres@alu.umh.es (S.T.); calabuix_sil@gva.es (S.C.-F.); fexposito@unav.es (F.E.); eredin@alumni.unav.es (E.R.); kvalencia@external.unav.es (K.V.); jantus_elo@gva.es (E.J.-L.); lmontuenga@unav.es (L.M.); 5 Molecular Oncology Laboratory, Fundación Hospital General Universitario de Valencia, 46014 Valencia, Spain; 6 TRIAL Mixed Unit, Centro de Investigación Príncipe Felipe-Fundación para la Investigación del Hospital General Universitario de Valencia, 46014 Valencia, Spain; 7 Department of Pathology, Universitat de València, 46010 Valencia, Spain; 8 DISNA and Program in Solid Tumors, Center for Applied Medical Research (CIMA), 31008 Pamplona, Spain; dserrano@unav.es; 9 Department of Pathology, Anatomy and Physiology, School of Medicine, University of Navarra, 31008 Pamplona, Spain; 10 Department of Biochemistry and Genetics, School of Sciences, University of Navarra, 31008 Pamplona, Spain; 11 Department of Biotechnology, Universitat Politècnica de València, 46022 Valencia, Spain

**Keywords:** epigenetic biomarkers, cancer, DNA methylation, micro-RNAs

## Abstract

**Simple Summary:**

Apart from genetic changes, cancer is characterized by epigenetic alterations, which indicate modifications in the DNA (such as DNA methylation) and histones (such as methylation and acetylation), as well as gene expression regulation by non-coding (nc)RNAs. These changes can be used in biological fluids (liquid biopsies) for diagnosis, prognosis and prediction of cancer drug response. Although these alterations are not widely used as biomarkers in the clinical practice yet, increasing number of commercial kits and clinical trials are expected to prove that epigenetic changes are able to offer valuable information for cancer patients.

**Abstract:**

Early alterations in cancer include the deregulation of epigenetic events such as changes in DNA methylation and abnormal levels of non-coding (nc)RNAs. Although these changes can be identified in tumors, alternative sources of samples may offer advantages over tissue biopsies. Because tumors shed DNA, RNA, and proteins, biological fluids containing these molecules can accurately reflect alterations found in cancer cells, not only coming from the primary tumor, but also from metastasis and from the tumor microenvironment (TME). Depending on the type of cancer, biological fluids encompass blood, urine, cerebrospinal fluid, and saliva, among others. Such samples are named with the general term “liquid biopsy” (LB). With the advent of ultrasensitive technologies during the last decade, the identification of actionable genetic alterations (i.e., mutations) in LB is a common practice to decide whether or not targeted therapy should be applied. Likewise, the analysis of global or specific epigenetic alterations may also be important as biomarkers for diagnosis, prognosis, and even for cancer drug response. Several commercial kits that assess the DNA promoter methylation of single genes or gene sets are available, with some of them being tested as biomarkers for diagnosis in clinical trials. From the tumors with highest incidence, we can stress the relevance of DNA methylation changes in the following genes found in LB: *SHOX2* (for lung cancer); *RASSF1A*, *RARB2*, and *GSTP1* (for lung, breast, genitourinary and colon cancers); and *SEPT9* (for colon cancer). Moreover, multi-cancer high-throughput methylation-based tests are now commercially available. Increased levels of the microRNA miR21 and several miRNA- and long ncRNA-signatures can also be indicative biomarkers in LB. Therefore, epigenetic biomarkers are attractive and may have a clinical value in cancer. Nonetheless, validation, standardization, and demonstration of an added value over the common clinical practice are issues needed to be addressed in the transfer of this knowledge from “bench to bedside”.

## 1. Introduction

Aberrant epigenetic changes are recognized as one of the key events leading to carcinogenesis [1]. Cancer cells harbor global epigenetic abnormalities in addition to genetic alterations. The use of “omic” techniques in recent years has allowed us to get a comprehensive view of the extensive reprograming that occurs in the epigenetic machinery of cancer cells. These epigenetic changes include DNA methylation, histone modifications, nucleosome positioning, and de-regulation of non-coding RNAs, mainly micro-RNAs (miRNAs) [2].

The most widely studied epigenetic modification and the one closer to be transferred to the clinic as a cancer biomarker is DNA methylation. This modification is the result of the addition of a methyl group at the 5′-carbon of the pyrimidine ring of a cytosine followed by a guanine (CpG), which impedes gene transcription. Cancer is characterized by global DNA hypomethylation and focal hypermethylation of certain genes such as tumor suppressor genes [3] or miRNAs, whose silencing promotes tumor growth [4]. Hypomethylation takes place mainly in repetitive regions of the genome and has been shown to facilitate genomic instability and DNA damage [5].

**Figure 1:**
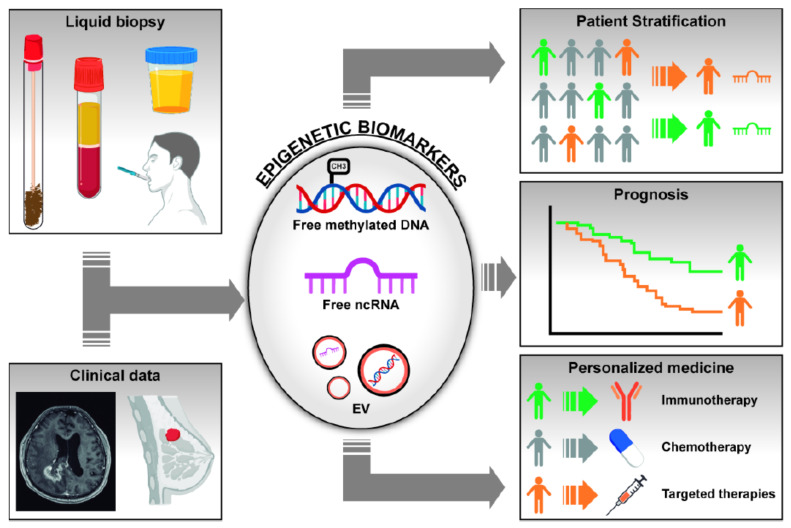
Scheme representing the utility of epigenetic changes found in liquid biopsies as biomarkers for cancer diagnosis, patient’s stratification, prognosis, and response to treatments. Changes in DNA methylation of gene promoters and abnormal levels of non-coding RNAs (ncRNAs) can be found in fluids as free molecules or inside extracellular vesicles (EVs). Integration of these biological markers with clinical and radiological data may help in the management of cancer patients, in particular in the field of screening and diagnosis.

Both genetic and epigenetic alterations identified in cancer can be used as biomarkers for diagnosis, prognosis, and prediction of drug response. Although assessment of biomarkers in tumor specimens may offer direct information about genetic and epigenetic alterations, the amount of tissue obtained from advanced tumors is often insufficient and may not reflect the whole tumor heterogeneity. To overcome these inconveniences, an alternative option to tissue samples has emerged in the last years, known as liquid biopsy (LB). LB is an non-invasive method that allows for the analysis of different biomarkers in fluids such as blood, saliva, bronchoalveolar lavage (BAL), cerebrospinal fluid (CSF), urine, or other body fluids [6]. These samples are easily obtained and may pick up DNA/RNA/proteins coming from both the primary tumor and the different metastatic sites, representing tumor heterogeneity and clonal evolution. In LB, we can find circulating tumor DNA (ctDNA), circulating tumor RNA (ctRNA), circulating tumor cells (CTCs), and extracellular vesicles (EVs) that may contain RNA, proteins, and DNA. Figure 1 graphically depicts the possible contribution of epigenetic biomarkers (free or vesicle-enclosed methylated DNA and ncRNAs) isolated in LB, in conjunction with clinical data, for patient’s stratification, prognosis, and prediction of response to therapy [6].

The clinical value of identifying actionable genetic mutations in LB (mainly blood) to treat patients with targeted therapy has been widely proven. However, regarding epigenetic changes, translation of these potential biomarkers into the clinic still lags far behind the genetic biomarkers. Although with some exceptions, rather than prediction of response to drugs, epigenetic biomarkers could be particularly useful as diagnostic and prognostic indicators, with numerous commercially available tests already developed to detect changes in DNA methylation levels [7]. The performance of diagnostic test is commonly evaluated in terms of sensitivity, specificity, and the area under the ROC curve (AUC). Sensitivity is defined as the percentage of positive cases that is correctly identified and specificity as the percentage of negative cases that is correctly identified. The AUC, which takes into account both sensitivity and specificity, defines diagnostic accuracy and is optimal when values are closer to 1. The fact that epigenetic changes are found early in carcinogenesis and that DNA methylation is stable in ctDNA, makes this epigenetic modification an excellent potential cancer diagnostic biomarker in LB.

**Figure 2:**
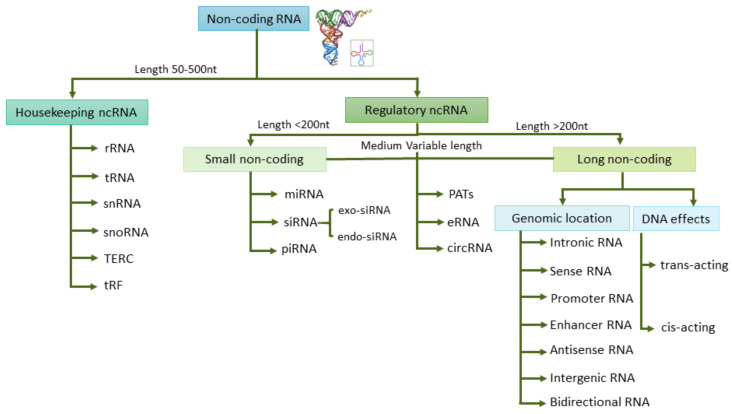
Non-coding (nc)RNAs classification into different groups based on their length and their regulatory roles. Small non coding RNA (sncRNA), long non coding RNA (lncRNA), ribosomal RNA (rRNA), transfer RNA (tRNA), small nuclear (snRNA), small nucleolar (snoRNA), telomerase RNA component (TERC), tRNA-Derived Fragments (tRF) and tRNA halves (tiRNA), microRNA (miRNA), small interfering RNA (siRNA), piwi-interacting RNA (piRNA), promoter-associated transcripts (PATs), enhancer RNA (eRNA), circular RNA (circRNA), and long non-coding RNA (lncRNA).

The term “epigenetic”, considered as any change in gene expression that does not permanently affect the DNA, may also include gene expression regulation by non-coding (nc)RNAs and histone modifications [8]. The aberrantly expressed ncRNAs may be promising therapeutic targets as well as cancer biomarkers. ncRNAs are the principal regulators of key molecular and cellular processes such as RNA splicing, gene regulation, proliferation, and apoptosis. ncRNAs can be classified into two groups based on their length and their roles: housekeeping ncRNAs and regulatory ncRNAs, which in turn include small ncRNAs and long ncRNAs (Figure 2). Circulating RNA species can be found free in fluids or inside EVs, where they are protected from degradation. EVs can be classified into three main types according to their size and biogenesis: exosomes, microvesicles, and apoptotic bodies [9]. Approximately 70% of studies have assessed exosomes as the source of choice for ncRNA when evaluating biomarkers [9]. Yuan et al. analyzed the RNA content in exosomes and estimated that mature miRNAs spanned 40.4%, piwi-interacting RNAs 40%, pseudo-genes 3.7%, lncRNAs 2.4%, tRNAs 2.1%, and mRNAs 2.1% of the total RNA [10]. From the different species of ncRNAs, microRNAs stand out as potential epigenetic markers in fluids, although implementation in the clinic encounters several difficulties such as RNA instability and variability of the methodologies used [11]. In general, RNA is less stable than DNA and proteins, and in particular, some regulatory lncRNAs show short half-lives [12]. Besides, there are many factors that may influence RNA stability in body fluids such as hemolysis in plasma/serum samples, which are a major cause of variation in miRNA levels [13]. Another possible pitfall when analyzing tumor miRNAs in liquid biopsy is their unknown cellular origin and the masking effect from ncRNAs released by non-tumor cells. Several studies postulate that the material contained in exosomes derived from the tumor microenvironment (TME) can also contribute to the characterization of the tumor, and as a consequence, TME-derived exosomes could be a good source for biomarkers [14].

Changes in histone modification have also been identified in circulation in cancer patients and are another source of epigenetic biomarkers. Nonetheless, due to the complexity of modifications, we will not cover it in our review. Information on this issue has been comprehensively reviewed in a recent study [15].

In this review, we address the most relevant evidence (according to authors’ criteria) on epigenetic biomarkers in LB, with special emphasis on tumors with high incidence. We summarize the data about biomarkers currently registered on the market as well as novel emerging candidates. 

## 2. Types of Biological Fluids for Epigenetic Analysis

In cancer patients, ctDNA can harbor the same mutational and epigenetic traits as the corresponding tumor [16]. A common and convenient source of LB is blood, but certain tumors are characterized by shedding low amounts of DNA into the blood (i.e., brain, kidney, bladder, prostate, thyroid, or head and neck cancers). In these cases, since their tumor location allows a direct communication with other body fluids, it could be more informative to use alternative samples for the analysis of biomarkers [17].

In head and neck cancer, saliva is an attractive non-invasive sample for screening, diagnosis, and monitoring due to its simple collection and low cost. Salivary nucleic acids have been used, for example, for the identification and validation of DNA methylation and miRNAs, demonstrating their utility in several clinical contexts (recently reviewed by [18]). Airway-derived fluids such as bronchial aspirates/lavages and sputum samples, have proven to be accurate tools for the early detection of tumors arising in the respiratory system [7]. Pleural effusion is also a very informative biological sample for biomarker assessment in lung cancer (LuCa) patients. It is well established that the *EGFR* mutational status can be reliably determined in ctDNA from pleural effusions to predict response to EGFR-tyrosine kinase inhibitors (TKIs) [19]. In contrast, the information on epigenetic biomarkers in pleural fluid is more limited and the clinical value of such biomarkers has to be clearly determined, but recent reports are finding possible association with prognosis [20].

Urine is a bona-fide source of epigenetic biomarkers in the case of genitourinary cancers. DNA hypermethylation has been described as one of the earliest and most frequent aberrations in prostate cancer (PrCa), and the detection of methylation patterns in urinary ctDNA has shown to be clinically meaningful [21]. In bladder cancer (BdCa), promising results from urine-based tests that measure DNA methylation patterns have been described [22]. The potential of miRNAs as biomarkers (individually or in combination) has also been demonstrated for BdCa, showing high sensitivity and specificity [23].

In the case of central nervous system (CNS) tumors and due to the existence of the blood–brain barrier, ctDNA in CSF seems to be a better source than blood [24]. A high concordance between methylation patterns in CSF and matched tumor samples has been reported, indicating the potential use of this biofluid for epigenetic biomarker analysis [22,25].

## 3. Technologies for Epigenetic Assays in Liquid Biopsy

Several techniques have been described for the analysis of epigenetic alterations in CTCs, free circulating nucleic acids and exosomes [7,26,27]. According to the number of targets analyzed, these technologies can be divided into (a) single-locus or multiplexed assays; and (b) genome-wide approaches, which are mainly based on microarrays and next-generation sequencing (NGS).

### 3.1. DNA Methylation

The analyses of DNA methylation in ctDNA by single-locus or multiplexed assays are mainly amplification-based methods such as methylation specific PCR (MSP) or digital droplet PCR (ddPCR). MSP is a classical method that encompasses Methylight and MethylQuant assays. MSP detects a small amount of ctDNA among a considerable number of circulating free DNA (cfDNA) [23]. In Methylight assays, the DNA methylation level is analyzed by comparing the fluorescence of specific probes between methylated and unmethylated molecules [28,29]. ddPCR is an ultrasensitive and quantitative method that is useful for the discovery of clinical biomarkers in samples with a low amount of cfDNA. This method is based on a PCR that is conducted in water-oil emulsion droplets where a single DNA molecule can be amplified inside each droplet, thus avoiding the mask effect of the non-target DNA [26,28]. Moreover, there are other approaches that combine techniques used for ctDNA studies such as BEAming technology, epityper epigenetic analysis, and methylation sensitive high-resolution technology (MS-HRT). BEAming technology is a method that combines ddPCR and flow cytometry for the analysis of ctDNA [30,31]. Epityper epigenetic analysis combines specific enzymatic cleavage with mass spectrometry (MALDI-TOF-MS) [32]. MS-HRT compares the melting profiles of sequences that present differences in their base compositions [7,26].

Regarding genome-wide assays, the methylation analysis of ctDNA can be performed by different techniques such as Infinium DNA methylation EPIC array (EPIC). EPIC is considered the gold standard method for DNA methylation assays due to its cost-effectiveness and its ability to examine more than 850,000 CpG sites [33].

### 3.2. Non-Coding RNAs

The expression of ncRNAs can be evaluated in LB at targeted-specific level using amplification-based methods such as reverse transcription quantitative PCR (RT-qPCR) and ddPCR. Of note, the use of ddPCR represents a highly sensitive method to quantify the expression of specific transcripts in LB [34,35,36]. In addition, there are other targeted methods such as peptide nucleic acids (PNAs)-based fluorogenic biosensors [37] and the NanoString nCounter platform that allow for the detection of ncRNA expression levels without the need of previous amplification [38]. In particular, the NanoString nCounter platform is able to analyze a large panel of miRNAs in several types of biological fluids including plasma and urine [39]. Other targeted approaches have also been developed for the detection of miRNAs in CTCs such as in situ hybridization (ISH) with locked-nucleic-acid (LNA) probes [40] and methods based on signal amplification in microfluidic droplets for single-cell analysis of multiple miRNAs [41].

The expression of ncRNAs can also be detected in LB at transcriptomic level with the use of NGS (RNA-Seq) or microarrays. Although both technologies allow for the analysis of ncRNA transcripts in LB [42,43], unlike microarrays, RNA-Seq does not require prior knowledge of the target transcripts and shows higher sensitivity for the detection of ncRNAs [42,43]. A summary of the types of technologies, applications, and advantages/disadvantages can be found in Table S1.

## 4. Epigenetic Biomarkers in Lung Cancer

Lung cancer (LuCa) is currently the second most commonly diagnosed cancer and the leading cause of cancer-related deaths worldwide among women and men. Globally, there were an estimated 2.2 million lung cancer cases and 1.8 million deaths in 2018, accounting for approximately a third of all cancer cases and deaths [44]. LuCa is one of the most aggressive tumor types, with a 5-year survival rate that varies globally but remains consistently low, not exceeding 19% [45]. There are two main types of LuCa: non-small cell lung cancer (NSCLC, ~85% cases) and small cell lung cancer (SCLC, ~15% cases). NSCLC is subdivided in three main histological subtypes: adenocarcinoma (LUAD) (~40% of NSCLC cases), squamous cell carcinoma (LUSC) (~30% of NSCLC cases), and large-cell carcinoma (~10–15% of NSCLC cases).

Despite breakthroughs in LuCa treatments in the last few decades, which have gradually improved patient’s outcome, the mortality rate is still considerably behind that observed for other prevalent types such as breast or colon cancer. A major factor is the late diagnosis and, consequently, its late-stage presentation. In recent years, increased interest has been directed toward the use of imaging techniques and biomarkers for screening and early detection. In randomized trials, the use of low-dose computed tomography (LDCT) in populations at risk has shown a significant reduction in lung cancer mortality [46]. However, there are still several questions regarding LDCT, which require further research. Examples are clarification of cost-effectiveness in different populations; characterization of detected nodules with indeterminate risk level; the small but significant percentage of false-positive cases and the potential harms associated with unnecessary invasive interventions (biopsies or even surgeries) of these cases; and the potential tools to optimize risk assessment, to recommend for screening only those individuals with higher risk, not only based on age and smoking exposure [47]. It is then possible that the use of LB-based molecular biomarkers in screening programs might help LDCTs in identifying NSCLC. Biological fluids that can be used as a source for biomarkers in LuCa include blood, bronchial aspirates (BAS), bronchial lavages (BAL), sputum or pleural effusions, for analysis of ctDNA, exosomes, and CTCs.

### 4.1. DNA Methylation

Among DNA methylation biomarkers in LuCa diagnosis, *SHOX2* hypermethylation is clearly the best studied epigenetic alteration. *SHOX2* hypermethylation was first described by Schmidt et al. using bronchial fluid aspirates during bronchoscopy, showing 68% sensitivity and 95% specificity [48]. Other studies [49,50,51] have later validated the diagnostic potential of *SHOX2* methylation status in plasma and pleural effusions. The EpiProLung^®^ assay is the only commercial test specifically designed for LuCa diagnosis. This test is based on a PCR assay that analyzes methylation of *SHOX2* and *PTGER4* in blood, with a sensitivity of 78% and specificity of 96%, and an area under the ROC curve (AUC) of 0.73. This gene combination has also been tested in lavage fluid samples.

Other genes have been found to be differentially methylated in plasma samples when comparing LuCa patients and healthy controls including *DCLK1* (49% sensitivity and 91% specificity) [52], *SEPT9* (44% sensitivity and 92% specificity) [53], *RASSF1A* and *RARB2* (87% sensitivity and 75% specificity) [54]. Hulbert et al. demonstrated that analyzing the DNA methylation status of different genes such as *TAC1* (86% sensitivity and 75% specificity)*, HOXA7* (63% sensitivity and 92% specificity) and *SOX17* (84% sensitivity and 88% specificity) allowed for the detection of LuCa in sputum samples with a global sensitivity of 98% and specificity of 71% [55,56]. Interestingly this group has also published that methylation analysis of *CDO1*, *TAC1*, *HOXA9*, and *SOX17* in urine (as well as in plasma) can be useful as an adjunct to LDCT screening [57]. Recently, our group has developed an epigenetic model identified through epigenomic analysis by which the DNA methylation status of four genes (*BCAT1*, *CDO1*, *TRIM58*, and *ZNF177*) in BAS/BAL/sputum samples was able to discriminate between NSCLC patients (even at early stages) and controls (82% sensitivity and 76% specificity, AUC, ~0.9) [55,58]. We have also described that *TMPRSS4* hypomethylation can be used as a diagnostic tool in early stages, with an AUC of 0.72 (52% sensitivity and 91% specificity) for BAL and 0.73 (90% sensitivity and 65% specificity) for plasma [59].

Through genome-wide DNA methylation assays, Hsu et al. detected a multiple epigenetic panel in tumor samples by studying the methylation status of genes *CDH13*, *BLU*, *FHIT*, *RASSF1A*, and *RARB*, whose diagnostic potential was also validated in plasma samples with a sensitivity of 73% and a specificity of 82% [60]. Similarly, Ostrow et al. validated in plasma a group of four genes (*DCC*, *Kif1a*, *NISCH*, *RARB*) that was previously found in tumors, which discriminated between LuCa patients and tumor-free individuals, with a sensitivity of 73% and specificity of 71% [61]. In addition, Ooki et al. described a serum-based gene signature, previously identified in tumors from TCGA (*MARCH11*, *HOXA9*, *CDO1*, *UNCX*, *PTGDR*, and *AJAP1*) that was able to differentiate stage I NSCLC patients from the controls, with 72.1% sensitivity and 71.4% specificity [62].

Unlike for diagnosis, only a few studies have described the association between DNA methylation status and outcome or response to drugs using ctDNA [63,64]. Hypermethylation of *SHP1P2* in plasma was associated with reduced progression-free survival (PFS) in advanced NSCLC [65]. DNA methylation of the gene panel *SOX17*, *BMRS1*, and *DCLK1* in plasma had a negative impact on survival [52,66,67], whereas *SFN* methylation in serum samples was associated with improved survival [68]. Salazar et al. described that patients with unmethylated *CHFR* had an improved survival when treated with second-line EGFR TKIs [69]. Additionally, increased plasma ctDNA methylation levels of *RASSF1A* and *APC* within 24 h after chemotherapy administration was found to be associated with good response to cisplatin [70]. In addition, using plasma samples, prolonged survival has been observed in patients with low *SHOX2* promoter methylation after chemotherapy/radiotherapy [48].

### 4.2. ncRNAs

ncRNAs are also becoming a valuable tool for the early detection of LuCa. miRNAs, the most widely studied type of ncRNA, provide promising biomarkers for the diagnosis and prognosis of LuCa [71]. For instance, miR-1285 was significantly decreased while miR-324-3p was significantly increased in plasma of stage I LUSC patients in contrast to healthy donors (AUC 0.85 and 0.79, respectively) [72]. Chen et al. described 10 miRNAs (miR-20a, miR-222, miR-221, miR-320, miR-152, miR-145, miR-223, miR-199a-5p, miR-24, miR-25) able to discriminate NSCLC patients from healthy controls, with high sensitivity (92.5%) and specificity (90%) rates (AUC 0.97) [73]. miR-21, the most commonly studied miRNA in LuCa, has been found consistently upregulated in both serum and plasma samples and may serve as a diagnostic biomarker of early-stage NSCLC. Yu et al. reported that miR-21 was suitable for diagnosis, with 69% sensitivity and 71.9% specificity [74]. A multicenter study was performed with a total of 3102 participants to investigate the potential use of circulating miRNAs as diagnostic biomarkers in LuCa. Results reported that a 14-miRNA signature might be useful to discriminate patients with early-stage lung cancer (stage I or II) from healthy individuals. Specifically, miR-374b-5p differentiated patients with early-stage LuCa from those without cancer, with an AUC of 0.83 [75]. Some groups have studied miRNA precursors as diagnostic biomarkers in LuCa. Powrózek et al. reported that miRNA-944 precursors distinguished SCC from ADC with 78.6% sensitivity and 91.7% specificity (AUC = 0.77), and pri-miRNA-3662 discriminated SCC from ADC with 57.1% sensitivity and 90% specificity (AUC = 0.845). Both markers allowed to distinguish stage I-IIIA NSCLC from healthy individuals with 75.7% sensitivity and 82.3% specificity (AUC = 0.898) [76].

Some ncRNAs have also been proposed as prognostic biomarkers for LuCa. A recent study using a cohort of 182 patients with resected early-stage NSCLC reported that, among 84 circulating microRNAs, only miR-126-3p had an independent prognostic value in SCC patients [77]. Moreover, Yanaihara et al. showed that high expression of precursor has-mir-155 could be an independent poor prognosis biomarker in ADC patients [78]. Increasing evidence shows that lncRNA can also act as biomarkers for prognosis. Xie et al. reported in a cohort of 460 patients that low serum levels of SOX2OT and ANRIL were associated with higher overall survival (OS) rate. Multivariate analysis revealed that SOX2OT could be an independent prognostic factor for NSCLC [79]. Yung-Hung Luo et al. studied the correlation between clinicopathological characteristics and circRNAs using plasma from a cohort of 231 LuCa patients (65 had stage I–II and 166 stage III–IV) and 41 healthy donors. They reported that higher levels of circ_0000190 were correlated with larger primary tumor size, advanced stage, extrathoracic metastasis, and poor survival [80].

miR-21 has been identified as a key miRNA in the regulation of acquired resistance to EGFR-TKIs in NSCLC, and high serum levels of this miRNA have been found to be significantly increased at the time of EGFR-TKI progression when compared to those observed before treatment [81]. Wang et al. also demonstrated that patients who were resistant to EGFR-TKIs had higher levels of circulating miR-21, miR-27a, and miR-218 than patients who were sensitive [82]. Jinshuo Fan et al. found that NSCLC patients who were responsive to ICIs (immune checkpoint inhibitors) had increased levels of a signature composed of miR-27a, miR-28, miR-34a, miR-93, miR-106b, miR-138-5p, miR-181a, miR-193a-3p, miR-200, and miR-424 compared to non-responders. Moreover, patients with high levels of this signature showed improved PFS and OS than those where levels were low [83]. Recently, expression of circ_0000190 has been found to be correlated with PD-L1 expression and response to immunotherapy in NSCLC [80].

## 5. Epigenetic Biomarkers in Genitourinary Cancers

The most prevalent tumors of the genitourinary (GU) tract are prostate cancer (PrCa), bladder cancer (BdCa), and renal cell carcinoma (RCC) [84]. PrCa is the second most commonly diagnosed cancer and the sixth leading cause of cancer death among men worldwide [85]. PrCa diagnosis has not evolved significantly since the 1980s, when blood levels of prostate-specific antigen (PSA) were first introduced as a follow-up marker for recurrent tumors, and, subsequently, for early detection in combination with digital rectal examination (DRE) [77,86]. PSA for PrCa screening has low positive predictive value (~30%), potentially driving to over-diagnosis and over-treatment. This highlights the need of more accurate biomarkers that are alternative or complementary to PSA for screening and diagnosis [87]. In the case of BdCa, there was an estimated number of 550,000 cases and 200,000 deaths (2.1% of all cancer deaths) in 2018 [88]. The 5-year survival rates (~77%) have remained mostly unchanged since the 90s [89]. Although less frequent, RCC accounts for ~2% of all diagnosed cancers, but its incidence has more than doubled over the past fifty years, being the tenth most common neoplasm in men [84,90]. RCC diagnosis is mostly an accidental finding and represents 1.8% of all cancer deaths worldwide [90]. The therapeutic options for RCC have increased tremendously in recent years, but biomarkers of response to these drugs are still lacking.

### 5.1. DNA Methylation

A common trait of GU tumors is the possibility of using urine as a biological fluid for the analysis of CTCs and ctDNA [7]. Commercial epigenetic-based kits for the detection of PrCa and Bdca in both urine and blood samples are currently available [7]. Unfortunately, epigenetic markers in liquid biopsies from RCC patients are underdeveloped, as this is one of the tumor types with less ctDNA shedding into biological fluids [91,92,93]. Among DNA methylation biomarkers in GU cancers, *GSTP1* hypermethylation is by far the most frequently described epigenetic alteration, especially in PrCa patients [94]. Many authors have described its utility for PrCa detection [95,96] showing much higher specificity (~90%) than PSA (~30%), although sensitivity was similar for both PSA levels and *GSTP1* methylation [87,95]. Matched assessment of ctDNA *GSTP1* in urine and plasma samples revealed that urinary analysis outperforms plasma for diagnostic purposes [96,97]. It is worth mentioning that the DNA methylation analysis of multi-gene panels in serum including *GSTP1*, *RASSF1A*, and *RARB* have increased the diagnostic coverage of *GSTP1* alone [98] for PrCa. Similar strategies have also been used for BdCa detection (with 100% sensitivity) in a multi-gene panel that assessed *CDKN2A, ARF, MGMT,* and *GSTP1* [86]. In the case of RCC, methylation analysis of serum cfDNA using *GSTP1* alone or in combination with either *APC, p14ARF, p16, RARB, RASSF1, TIMP3*, or *PTGS2* has been shown to provide a high accuracy of detection (AUC ranging from 0.73 to 0.75; 95% IC 0.50–0.84) [99]. Independently, Hoque et al. measured *GSTP1* together with *CDH1, APC, MGMT, RASSF1A, p16, RARB2*, and *ARF* methylation for RCC detection using urine and plasma samples, showing that at least one gene was hypermethylated in 88% and 67% of the patient’s urine sediments and plasma, respectively. [100].

Other gene panels that do not include *GSTP1* are also under study for PrCa and BdCa detection. Analysis of *MCAM, ERalpha*, and *ERbeta* showed 75% sensitivity and 70% specificity for early PrCa detection [101]. Similarly, *ST6GALNAC3, ZNF660*, *CCDC181*, and *HAPLN3* detected PrCa patients with up to 100% specificity and 67% sensitivity [102]. With respect to BdCa, methylation status of several genes are reliable alone or in combination using ctDNA in serum: *CDH13* [103], *PCDH10* [104], and *PCDH17* [105]. Additionally, dual combinations such as *PCDH17* and *POU4F2* (93.96% sensitivity, 90% specificity) [106] or *NID2* and *TWIST1* (90% sensitivity and 93% specificity) [107] have been proven to be accurate for the detection of BdCa patients using urine samples [108]. Several commercial tests that include epigenetic and non-epigenetic biomarkers are now available for the diagnosis of BdCa using urine or blood (AssureMDx^®^, Bladder CARE^®^, Bladder EPICHECK^®^). In RCC, a panel of genes that act as Wnt antagonists can serve as biomarkers for diagnosis, staging, and prognosis using serum ctDNA [109]. Notably, Vitale Nuzzo P et al. used cell-free methylated DNA immunoprecipitation and high-throughput sequencing (cfMeDIP–seq) as a highly sensitive assay capable of detecting and discriminating early-stage RCC from other tumor types and healthy controls in plasma (AUC 0.9) and urine (AUC 0.86) [110]. Taking into account the different studies related to diagnosis in PrCa, BdCa, and RCC, hypermethylation of *RASSF1A, APC, RARB2*, and *ARF* [111,112] seem to be the most consolidated biomarkers to use in plasma, serum, and/or urine.

The potential of DNA methylation analysis in LB related to progression and therapy response is an area of intense study. Sunami et al. reported that methylation of *GSTP1, RASSF1A*, and *RARB2* associated with PrCa’s Gleason score and serum PSA; in addition, *GSTP1* and *RARB2* were associated with the disease’s advanced stage [98]. Likewise, it has recently been shown that hypermethylation of *APC, GSTP1,* and *RARB2* in urine sediments correlated with shorter RFS and higher PrCa grade [113]. Additionally, in the case of urine, dual assessment of *GSTP1* and *APC* discriminates between low-risk and aggressive PrCa [114]. Interestingly, Zhao et al. showed that monitoring *GSTP1, APC, CRIP3*, and *HOXD8* methylation was useful for noninvasive prediction of PrCa aggressive disease in patients on active surveillance [115]. Indeed, the same group of authors developed a PrCa urinary epigenetic assay (ProCUrE^®^) with diagnostic and prognostic purposes based on the optimized measurement of *GSTP1* and *HOXD3* gene methylation [116]. In the case of BdCa, *PCDH10* and *PCDH17* hypermethylation were independent predictors of cancer survival and correlated with higher stage and grade [104,105], as described for *NID2* and *TWIST1*, which were able to discriminate between different patient’s BdCa grades [108]. Finally, a multigene panel useful for BdCa recurrence surveillance has been developed, which included *EOMES, HOXA9, POU4F2, TWIST1, VIM,* and *ZNF154* [117]. A number of registered clinical trials (in some cases using commercial tests) for screening or recurrence purposes have been initiated in the case of PrCa and BdCa (https://www.clinicaltrials.gov/ (accessed on 30 December 2020)) (Table S2).

### 5.2. ncRNAs

In terms of ncRNA, several studies have shown their possible role as biomarkers in LB. Yu et al. recently designed a 4-lncRNA panel of urinary biomarkers (UCA1-201, HOTAIR, HYMA1, and MALAT1) for the diagnosis of non-muscle invasive bladder cancer (NMIBC) [118]. This signature confirmed the presence of tumor in a validation cohort of 140 NMIBC patients. A different study identified a 7-miRNA panel providing high diagnostic accuracy in BdCa using urine samples (miR-7-5p, miR-22-3p, miR-29a-3p, miR-126-5p, miR-200a-3p, miR-375, and miR-423-5p) [119]. Urquidi et al. described a sensitivity of 87% and a specificity of 100% using a different 25-miRNA urine signature [120]. An interesting approach integrated the expression of the mRNA HYAL1 together with two miRNAs (miR-96 and miR-210) and one lncRNA (UCA1) in an urine diagnostic panel that achieved a sensitivity of 100% and a specificity of 89.5% [121]. Interestingly, the lncRNA UCA1 increases cisplatin resistance in BdCa [122]. In the case of PrCa, ncRNA profiling could be a powerful tool to complement PSA screening. A recent study found a robust diagnostic model in serum using two different miRNAs (miR-17-3p and miR-1185-2-3p) with an associated 90% sensitivity and 90% specificity [123]. Serum detection of PSA in combination with miR-103a-3p and let-7a-5p detected PrCa cases better than PSA alone [124]. Serum miR-106b, miR-141-3p, miR-21, and miR-375 have also been combined in a panel with AUC of 0.86 [125].

## 6. Epigenetic Biomarkers in Breast Cancer

Breast cancer (BrCa) is the most common neoplasm diagnosed in females worldwide, with an incidence of 11.7% of all women cancer cases [44]. Screening based on imaging is key for the early detection and better prognosis of this disease, with mammograms being the most frequent technique recommended. However, the breast cancer nodules do not always exhibit pathognomonic characteristics, which can prevent the radiologist from performing a biopsy, or in other cases, generate false positives. Other limitations of this technique include the possible cumulative radiation exposure and over-diagnosis [126,127,128]. For these reasons among others, the search for potential non-invasive biomarkers in BrCa is needed.

### 6.1. DNA Methylation

Multiple studies have explored epigenetic alterations in ctDNA from BrCa patients that could serve for diagnosis, prognosis, classification of BrCa subtypes, and prediction of response to therapies. One of the most frequently studied markers has been the hypermethylation of *RASSF1A* [129,130,131,132,133], which discriminates between healthy individuals and BrCa patients and acts as a poor prognosis indicator [134]. Moreover, this aberration predicts the response to tamoxifen or neoadjuvant chemotherapy [135]. Other methylated targets found in plasma from BrCa patients with a diagnostic value encompass *SOX17, CST6, APC, DAK-K, MASPIN, HIC-1, HIN-1, RARB, RARbeta2, GSTP1, BRCA1*, and *KIF1A* [32,129,130,131,132,136,137,138,139]. Among these targets, the hypermethylation of *RASSF1A, BRCA1, RARB*, and *RARB2*, in estrogen receptor+ (ER+) and progesterone receptor (PR+) breast tumors and plasmas, were validated as indicators of poor prognosis in at least two independent studies [140]. Furthermore, Fujita et al. showed that the simultaneous detection of *RASSF1A, RARB*, and *GSTP1* methylation (93% specificity) was strongly correlated with poor outcome [141]. *SOX17* methylation is another independent prognostic factor (HR: 4.737; 95% CI: 2.088–10.747) and its methylation status in ctDNA from plasma samples was found to correlate (70.9% concordance) with that observed in CTCs from matched BrCa patients [142,143]. *PITX2* hypermethylation in plasma has also been reported as another indicator of poor OS (HR: 3.4; 95% CI: 1.2–9.8) [144]. Interestingly, *PITX2* hypermethylation also predicted the response to anthracycline-based therapy [145].

In relation to ER status, Martinez-Galan et al. demonstrated methylation of *ER* and *ESR1* promoters in plasma from ER- patients [146]. In contrast, *PTPRO* methylation was found as a prognostic factor (HR: 3.66; 95% CI: 1.371–9.784) in ER+ positive BrCa patients but not in ER− [141]. Some gene panels have been designed to simultaneously analyze several gene methylation patterns in BrCa serum/plasma. For instance, Visvanathan et al. developed a panel of 10 genes including *RASSF1A*, whose methylation index predicted worse PFS (HR: 1.79; CI 95%: 1.23–2.60, and OS (HR: 1.75; 95% CI: 1.21–2.54) in metastatic BrCa patients [147]. Although evaluation of the methylation status in these genes is promising, there is currently only one commercial kit available specifically for BrCa, which tests for *PITX2* methylation in paraffin samples (Therascreen^®^, Qiagen, Frankfurt, Germany) [134].

### 6.2. ncRNAs

The potential value of ncRNAs as BrCa biomarkers in serum or plasma (either in exosomes or as cfRNA) has also been reported. Exosomal miR-21 has been widely shown to be a diagnostic biomarker, with sensitivity and specificity in pooled studies of ~75% and ~85%, respectively, and an AUC of 0.93 [148]. There are hundreds of studies proposing miRNAs as diagnostic and prognostic biomarkers in BrCa, but they need validation. We summarize here some recent relevant publications. In BrCa plasma samples, combination of four miRNAs (miR-1246, miR-206, miR-24, miR-373) distinguished BrCa from healthy individuals with 98% sensitivity, 96% specificity, and accuracy of 97% [149]. A panel composed of exosomal miR-142-5p, miR-320a, and miR-4433b-5p isolated from a BrCa patient’s serum differentiated patients from their control counterparts with 93.33% sensitivity, 68.75% specificity, and AUC of 0.83 [135]. Furthermore, the combination of miR-142-5p and miR-320a discriminated luminal A subtype from healthy donors with 100% sensitivity, 93.80% specificity, and AUC of 0.94. Interestingly, decreased expression of miR-142-5p and miR-150-5p were significantly associated with more advanced tumor grades (grade III), while the decreased expression of miR-142-5p and miR-320a was associated with a larger tumor size [135]. Additionally, circulating miR-30b-5b has been recently reported to act as a BrCa prognostic factor [150].

Serum miRNA profiles may be useful for the diagnosis of axillary lymph node metastasis before surgery in a less-invasive manner than sentinel lymph node biopsy. A model that includes a combination of two miRNAs (miR-629-3p and miR-4710) and three clinicopathological factors (T stage, lymphovascular invasion, and ultrasound findings) showed an optimal diagnostic potential, with 88% sensitivity, 69% specificity, and accuracy of 0.86 [151].

There are also data that correlate ncRNA levels in serum to treatment response. For example, a set of exosomal miRNAs (miR-185, miR-4283, miR-5008 and miR-3613, miR-1302, miR-4715, and miR-3144) that target pathways of immune maturation predicted poor neoadjuvant chemotherapy response prior to surgery [152]. Similarly, lncH19 levels in the plasma of BrCa patients have also been reported to predict response to neoadjuvant chemotherapy [153].

## 7. Epigenetic Biomarkers in Colorectal Cancer

Colorectal cancer (CRC) is the third most common cancer worldwide. This tumor represents approximately 10% of all diagnosed cancer cases, with approximately 1.8 million new cases estimated in 2018. It is important to note that CRC is responsible for approximately 9% of all cancer deaths, being the second leading cause of cancer mortality [154]. In CRC, screening strategies have been shown to be effective to detect early CRC and precancerous lesions, and to reduce its morbidity and mortality. Among the detection strategies, the fecal immunochemical test (FIT) represents a non-invasive and cost-effective assay for detecting the presence of fecal hemoglobin. This is currently the most commonly used method for CRC screening, with an overall sensitivity and specificity for detection of 79% and 94%, respectively. However, the ability of this assay to detect advanced precancerous lesions is limited, showing 24% sensitivity and 95% specificity [155]. After a positive result for FIT, colonoscopy is the gold standard diagnostic technique for CRC detection. However, it is an invasive method that needs bowel preparation and sedation, presenting certain risk of complications for the patients [156]. In this context, the use of epigenetic biomarkers such as DNA methylation in stool samples might provide a non-invasive and the most cost-effective approach in population-based screening for both CRC and precancerous lesions [157]. Thus, for example, the simultaneous methylation analysis of *SEPT9* and *SDC2* (ColoDefense^®^ test) in stool samples was able to obtain a sensitivity of 66.7% for advanced adenoma (AA) and 92.3% for CRC, with a specificity of 93.2% [158].

### 7.1. DNA Methylation

Among the most frequently studied epigenetic biomarkers in ctDNA for CRC, the methylation of *SEPTIN9* (*SEPT9*) stands out for screening and early detection [144,159,160]. The EpiproColon^®^ test was the first commercially available FDA-approved test for the detection of *SEPT9* methylation in plasma by real-time PCR [161,162]. In addition to blood samples, methylation of this gene has also been analyzed in stool, showing a 35.9% improvement in detecting pre-tumoral stages (AA) and 7.9% in identifying early CRC tumors, in comparison with the plasma test [163]. The use of ColoDefense^®^ in blood enabled the detection of AA and CRC, with an overall sensitivity of 88.9% and a specificity of 92.8% [164]. Similarly, other studies have proposed the analysis of the methylation of several genes in plasma as circulating epigenetic biomarkers able to discriminate between healthy controls and patients with AA or CRC [165,166]. In addition, approaches based on methylation microarrays [33] and NGS [167] have been used to identify epigenetic biomarkers in ctDNA for cancer detection.

Regarding prognosis, hypermethylation of the *P16* promoter in ctDNA has been associated with poor OS [168]. Additionally, hypermethylation of *HPP1* and *HLTF* indicates a poor prognosis and high mortality [169], and hypermethylation of *RARB* and *RASSF1A* was associated with the aggressiveness of the disease [170] in patients with CRC. Methylation of ctDNA can also be used to monitor tumor burden and evaluate the therapeutic response of patients [171,172], correlating better than classical biomarkers such as carcinoembryonic antigen (CEA) and carbohydrate antigen (CA)-19-9. For example, the analysis of the methylation status of the 2-gene panel *BCAT1/IKZF1* in plasma showed higher sensitivity for detecting CRC recurrence than CEA, with an odds ratio of 14.4 (95% CI: 5–39) and 6.9 (95% CI: 2–22), respectively [173]. Similarly, the plasma methylation of *SEPT9*, *DCC*, *BOLL*, and *SRFP2* showed stronger correlation with tumor burden than CEA and CA-19-9 [172].

### 7.2. ncRNAs

Circulating levels of ncRNAs have also shown utility as biomarkers in the management of CRC. Circulating miR-21 levels in blood and saliva allow for the detection of CRC [174,175]. In addition, miRNA signatures evaluated in fluids can be useful for discriminating between healthy controls, patients with adenomas, and patients with CRC with high sensitivity and specificity. In particular, the plasma levels of miR-601 and miR-760 showed an AUC of 0.68 with 72.1% sensitivity and 62.1% specificity, which can discriminate between AA and healthy donors. In addition, this panel of miRNAs was able to differentiate CRC from the control samples with an AUC of 0.79, a sensitivity of 83.3%, and a specificity of 69.1% [176]. Another study has recently identified a signature of six miRNAs (miRNA19a, miRNA19b, miRNA15b, miRNA29a, miRNA335, and miRNA18a) with an AUC of 0.92, a sensitivity of 85%, and a specificity of 90% that is able to detect CRC and AA in comparison to healthy individuals [157]. Regarding prognosis, high levels of circulating miR-210 and miR-141 are associated with shorter survival [165,177], while high levels of miR-23b are associated with longer survival [178]. Besides, levels of different miRNAs in blood may be useful for the early detection of recurrence [179] and evaluation of therapy response in CRC patients [180]. High plasma levels of the lncRNA HOTAIR have shown utility for the detection of CRC and association with a worse prognosis and higher mortality [181]. Of note, other studies have analyzed different combinations of circulating lncRNAs as diagnostic biomarkers, which were useful for the detection of adenomas and CRC [166,182,183].

## 8. Epigenetic Biomarkers in Other Tumor Types and Multi-Cancer Tests

In addition to common tumor types, epigenetic alterations may also be detected in LB from other less frequent malignancies, showing clinical utility as tumor biomarkers. In cutaneous melanoma, where the use of circulating epigenetic biomarkers has been proposed as a non-invasive tool for tumor detection, promoter hypermethylation of *RASSF1A* has been described in plasma samples as a diagnostic indicator, with the ability of discriminating between melanoma patients and healthy individuals, showing a good diagnostic accuracy with an AUC of 0.90 [184]. Besides, the detection of hypermethylated *RASSF1A* in serum before treatment was able to predict the prognosis and clinical response to drugs in advanced melanoma patients [185]. In a recent pilot study using NGS and machine learning, Bustos et al. were able to identify a circulating miRNA signature (miR-4649-3p, miR-615-3p, and miR-1234-3p) associated with the response to ICIs in advanced melanoma patients, suggesting that circulating miRNAs could enable real-time monitoring of patients receiving this type of treatment [167]. The plasmatic levels of other ncRNAs such as lncRNAs (IGF2AS, anti-Peg11, MEG3, Zeb2NAT) were also found to be associated with prognosis and therapy response in *BRAF*-mutant advanced melanoma patients treated with the BRAF inhibitor vemurafenib [186]. Similar to melanoma, the blood-based analysis of DNA methylation and ncRNAs has shown utility as circulating epigenetic biomarkers for other tumors including pancreatic cancer [187], ovarian and endometrial carcinomas [188,189], and brain tumors [190], among others.

In brain tumors such as glioblastoma, promoter hypermethylation of several genes (*MGMT*, *p16INK4a*, *TIMP-3*, and *THBS1*) has been detected at high frequencies in serum and CSF. In glioblastoma, hypermethylation of *MGMT* is associated with response to temozolamide [191]. The methylation status of *MGMT* and *THBS1* in CSF was also able to independently predict PFS of glioblastoma patients [22]. Similar to methylation, the circulating microRNA profiling of CSF has also been proposed as a good approach for the non-invasive detection (miR-30e, miR-140, let-7b, mR-10a, and miR-21-3p) and prognosis (miR-10b and miR-196b) of glioblastoma patients [192]. In other tumor types such as oral cancer, the analysis of epigenetic biomarkers in saliva has been explored. In this sense, the promoter hypermethylation of different types of genes (e.g., *RASSF1A*, *p16 INK4a*, *TIMP3*, and *PCQAP*/*MED15*) and the expression levels of miRNAs in saliva have been detected in association with oral tumors [18,193]. Thus, the study of epigenetic biomarkers in saliva has been proposed as an easily accessible LB sample for oral cancer detection.

The recent application of NGS has allowed for the development of sensitive epigenetic assays for the detection of both common and less-frequent tumors. Thus, using NGS and machine learning, Liu et al., in a very large clinical trial including individuals with (n = 2482) and without cancer (n = 4207), recently developed a classifier based on the methylation of cfDNA, assessing >100,000 methylation sites in plasma for the sensitive detection of more than 50 tumor types [169]. This multi-cancer approach was useful across all stages of the disease, and also for the identification of the tissue of origin with high accuracy, which could be relevant for the treatment and follow-up of the patients. The assay is going to be commercialized by the Biotech Company GRAIL. PanSeer^®^ is an NGS-based assay that is able to detect cancer in asymptomatic individuals, years before standard diagnosis [194].

Table 1 shows a list of the top methylated genes and ncRNAs identified in LB from cancer patients, with an emerging role as biomarkers. This list has been established based on the number of studies and robustness of the genes/ncRNAs published and/or inclusion in commercial tests.

## 9. Epigenetic Biomarkers in Cancer: Translation to the Clinic

The use of non-invasive epigenetic biomarkers is considered as a promising option in oncology. However, these biomarkers (with few exceptions) have not successfully reached clinical practice yet. Progress in the path to translation will be made provided clinical value is added to the current management of patients. These are some of the difficulties to take into consideration for clinical translation [195]:

(1) Clinical value and confirmatory results: confirmed clinical evidence in prospective trials is critical for medical professionals and regulatory agencies.
(2) Performance and affordability: it is essential to develop a commercial product with demonstrated good performance, affordable price, and is easy to use.
(3) Pre-analytical issues: preservation of the sample, storage time, and temperature, etc., have to be extensively studied.
(4) Technical barriers: when using some of the epigenetic techniques, there may be a technical barrier, particularly for advanced procedures such as mass spectrometry or next generation sequencing (NGS).
(5) Training: formation on new epigenetic platforms and interpretation of the results is needed, especially for the “omic” epigenetic technologies.
(6) Global regulation: establishing a global harmonization of regulation would facilitate translating an epigenetic assay into the clinic.

Overall, the continuous technological development and commercialization activity of epigenetic kits would lead to an innovative and competitive environment that will result in significant benefits for the clinical practice in the near future.

## 10. Conclusions and Future Perspectives

Evaluation of epigenetic biomarkers in LB is an emerging field in oncology that may help in cancer screening, diagnosis, identification of tumor subtypes as well as in the prediction of response to therapy and outcome. LB offers the opportunity of evaluating tumor markers using non-invasive methods and may represent better tumor heterogeneity and evolution. While the evaluation of actionable mutations in LB has a demonstrated clinical value, the use of epigenetic alterations (with few exceptions) has not reached clinical practice yet. Among the different fields where epigenetic changes may play a role as biomarkers, we envision that screening and diagnosis are the areas closer to the clinic. Current screening tests such as mammography, analysis of occult blood in feces and colonoscopy are routinely performed to detect BrCa and CRC, respectively. Nonetheless, over-diagnosis and false positives are of concern. In the case of PrCa, blood levels of PSA lack diagnostic accuracy and for LuCa, LDCT is not a common practice yet. Therefore, epigenetic biomarkers in LB could be of great value in screening and diagnosis for these cancer types. Moreover, the development of platforms that analyze thousands of methylation alterations in blood has been shown to be highly valuable in screening for multiple cancers.

Some epigenetic commercial tests have been developed and are currently being evaluated in clinical trials. These tests are designed for individual cancer types or as multi-cancer diagnostic tools; some others include both DNA methylation and mutational assays in the same kit. With constant information being provided by “omic” techniques for both DNA methylation and ncRNAs, new potential sources of epigenetic markers will be introduced and tested. However, the path to clinical translation is long and costly and thus the identified epigenetic biomarkers need to offer an added value over the established clinical practice and to attract investment for their development.

The discovery of new gene/signature candidates can also face several issues. For example, in the case of blood, studies show the need to use large amounts of plasma or serum to evaluate DNA methylation (1–4 mL) in comparison with protein-based techniques that can use much lower amounts (10–100 μL). This limitation could be solved with the introduction of new ultrasensitive techniques. The discovery of novel aberrantly methylated genes using “omic” platforms may also need specialized technicians and bioinformaticians to analyze the data correctly. In addition, these technologies are expensive and could be outsourced at reference hospitals.

## Figures and Tables

**Table 1:** Top methylated genes/signatures and ncRNAs identified in liquid biopsies from cancer patients, with an emerging role as biomarkers. BAL: bronchoalveolar lavage; BAS: bronchoalveolar aspirate; CSF: cerebrospinal fluid.

Epigenetic Alteration	Gene Name(s)/Epigenetic Kit	Type of Liquid Biopsy	Intended Use	Reference
LUNG CANCER				
DNA methylation	*-SHOX2/PTGER4* (EpiProLung)^®^ -Gene sets including *RASFF1A* and other genes -*BCAT1*/*CDO1*/*TRIM58*/*ZNF177*	Blood	Diagnosis	[66]
		BAL/sputum	Diagnosis	[54,55,56,57,62]
		BAS/BAL/sputum	Diagnosis	[58]
ncRNAs	-miR21	Blood	Diagnosis	[74]
	-Several miRNA signatures	Blood	Diagnosis	[72,73]
GENITOURINARY CANCERS				
DNA methylation	-Gene sets including *GSTP1, RASFF1A, APC, ARF* and *RARB2* -Several gene sets (AssureMDx^®^, Bladder CARE^®^, Bladder EPICHECK^®^) -*GSTP1* and *HOXD3* (ProCUrE, Prostate cancer)	Urine, blood	Diagnosis	[94,96]
		Urine, blood	Diagnosis	[103,108]
		Urine	Diagnosis	[116]
ncRNAs	-Several miRNA and lncRNA signatures	Blood	Diagnosis	[118,119,120]
BREAST CANCER				
DNA methylation	-*Gene sets including GSTP1, RASFF1A, BRCA1 and RARB2* -*PITX2*	Blood Blood	Diagnosis Prognosis/response	[129,130,131,132,133,136,137,138,139] [144,145]
ncRNAs	-miR21 -Several miRNA signatures	Blood	Diagnosis	[148]
		Blood	Diagnosis	[149,151]
COLORECTAL CANCER				
DNA methylation	-*SEPT9* (EpiProColon^®^)	Stool, blood,	Diagnosis	[161,162]
	-*SEPT9 and SDC2* (ColoDefense^®^)	Stool, blood	Diagnosis	[158]
	-*p16, RASFF1A, RARB2*	Blood	Diagnosis, prognosis	[168,169,170,171]
	-*BCAT1* and *IKZF1*	Blood	Diagnosis	[172]
ncRNAs	-miR21	Blood, saliva	Diagnosis	[174,175]
	-Several miRNA signatures	Blood	Diagnosis	[176,177]
OTHER CANCER TYPES AND MULTI-CANCER BIOMARKERS				
DNA methylation	-*RASFF1A* (melanoma)	Blood	Diagnosis	[184]
	-*MGMT* (glioblastoma)	CSF	Response to therapy	[191]
	-*RASFF1A, p16, TIMP3* (oral cancer)	Blood, saliva	Diagnosis, prognosis	[193]
	-DNA methylation signature PanSeer^®^	Blood	Diagnosis	[194]
	-DNA methylation signature GRAIL^®^	Blood	Diagnosis	[169]
ncRNAs	-Several miRNA signatures	Blood	Diagnosis	[186,187,188,189]

## Data Availability

Non-applicable.

## Supplementary Materials

The following are available online at https://www.mdpi.com/article/10.3390/cancers13123016/s1, Table S1: Technologies for epigenetic assays in liquid biopsy, Table S2: Clinical trials using epigenetic biomarkers in PrCa, BdCa and RCC.
