# Supplementary material for: Cancer Epigenetic Biomarkers in Liquid Biopsy for High Incidence Malignancies
Source: Cancers (Basel). 2021 Jun 16;13(12):3016. doi: 10.3390/cancers13123016 (PMC8233712; doi:10.3390/cancers13123016)
Supplement: Supplementary file 1 [file cancers-13-03016-s001.zip › cancers-1180520-supplementary.pdf]

Supplementary Materials:

Cancer Epigenetic Biomarkers in Liquid Biopsy for High Incidence Malignancies

Table S1. Technologies for epigenetic assays in liquid biopsy.

Name	Nucleic Acid	Principle	Coverage	Sensitivity	Key Points	References
MSP	DNA	Specific primers annealing with modified or unmodified molecules of DNA after bisulfite conversion	Few CpGs	High	Cost-effective<br>Easy clinical application<br>Analysis by PCR	[1,2]
Methylight	DNA	Specific fluorescent probes that recognize modified or unmodified molecules of DNA after bisulfite conversion.	Few CpGs	High	Easy clinical application.<br>Analysis by qPCR	[3]
ddPCR	DNA/ ncRNA	The sample is divided into droplets. In each drop a unique molecule is amplified	Few CpGs	Very high	Great potential for clinical application in low-quantity samples	[2,4–6]
BEAMing	DNA	Emulsion-PCR and flow cytometry analysis	Few CpGs	Very high	Useful for diagnosing and monitoring disease	[7,8]
Epityper	DNA	Simultaneous detection of multiple target CpG residues by using thymidine-specific cleavage mass array on matrix-assisted laser desorption/ionization time-of-flight silicon chips	Few CpGs	Mid	Determines the DNA methylation percentage for each CpG site individually	[2,9]
MS-HRT	DNA	Uses the difference in melting temperature between methylated and unmethylated molecules after bisulfite conversion	Few CpGs	High	High reproducibility<br>Cost-effective<br>Analysis by qPCR	[2,10]
Microarrays	DNA/ ncRNA	Solid matrix with probes where DNA/cDNA hybridizes. Probe-target hybridization is detected and quantified by fluorescence or chemiluminescence	Genome-wide	Mid	Useful for discovering new biomarkers or classifying groups	[11–13]
PNAs-based fluorogenic biosensors	ncRNA	PNAs hybridizes with target miRNA and catalyze a fluorogenic reaction	Targeted	High	Cost-effective<br>Enzyme-free<br>Minimal sample processing	[14]
NanoString nCounter	ncRNA	Quantification of miRNA expression by hybridization to target specific color-coded probes	Targeted	High	No reverse transcription or amplification required	[15,16]
miRNA in situ hybridization	ncRNA	Labeled LNA probes hybridize to miRNA sequences. The probes can be identified via antibodies that are labeled with enzymes	Targeted	High	Single-cell analysis. CTCs	[17]

		that convert fluorogenic enzymatic substrates into fluorescent products				
Signal amplification in microfluidic droplets	ncRNA	Water-in-oil droplets for single cells encapsulation, isothermal amplification of miRNA and fluorescence detection	Targeted	High	Multiple miRNAs detection at single-cell level. CTCs	[18]
RNA-seq	ncRNA	NGS technique that allows the profiling of transcriptional expression	Genome-wide	High	It does not require knowledge of target ncRNAs<br><br>High-throughput	[12,13]

Table S2. Clinical trials using epigenetic biomarkers in PrCa, BdCa and RCC. Search terms: PrCa / BdCa / RCC AND Detection OR Recurrence OR Prediction.

NCT Number	Cancer Type	Setting	Study Type
NCT03494803	PrCa	Detection	Observational
NCT02657863	PrCa	Detection	Observational
NCT00340717	PrCa	Detection	Observational
NCT00977457	PrCa	Recurrence	Interventional
NCT01441687	PrCa	Prediction of biopsy outcome	Interventional
NCT04314245	BdCa	Detection	Observational
NCT03122964	BdCa	Diagnostic Test: AssureMDx	Observational
NCT00962052	BdCa	Biomarker discovery	Observational
NCT02647112	BdCa	Recurrence: Test EpiCheck	Interventional
NCT02745301	BdCa	Detection	Observational
NCT02700464	BdCa	Detection: Test EpiCheck	Interventional
NCT03563443	BdCa	Diagnostic Test: Genomic Imprinting Testing	Observational

References

[1] Shivapurkar, N.; Gazdar, A. F. DNA Methylation Based Biomarkers in Non-Invasive Cancer Screening. *Curr Mol Med*, **2010**, *10* (2), 123–132. https://doi.org/10.2174/156652410790963303.

[2] Diaz-Lagares, A.; Mendez-Gonzalez, J.; Hervas, D.; Saigi, M.; Pajares, M. J.; Garcia, D.; Crujerias, A. B.; Pio, R.; Montuenga, L. M.; Zulueta, J.; et al. A Novel Epigenetic Signature for Early Diagnosis in Lung Cancer. *Clin Cancer Res*, **2016**, *22* (13), 3361–3371. https://doi.org/10.1158/1078-0432.CCR-15-2346.

[3] Eads, C. A.; Danenberg, K. D.; Kawakami, K.; Saltz, L. B.; Blake, C.; Shibata, D.; Danenberg, P. V.; Laird, P. W. MethyLight: A High-Throughput Assay to Measure DNA Methylation. *Nucleic Acids Res*, **2000**, *28* (8), E32. https://doi.org/10.1093/nar/28.8.e32.

[4] Han, X.; Wang, J.; Sun, Y. Circulating Tumor DNA as Biomarkers for Cancer Detection. *Genomics Proteomics Bioinformatics*, **2017**, *15* (2), 59–72. https://doi.org/10.1016/j.gpb.2016.12.004.

[5] Solé, C.; Tramonti, D.; Schramm, M.; Goicoechea, I.; Armesto, M.; Hernandez, L. I.; Manterola, L.; Fernandez-Mercado, M.; Mujika, K.; Tuneu, A.; et al. The Circulating Transcriptome as a Source of Biomarkers for Melanoma. *Cancers (Basel)*, **2019**, *11* (1). https://doi.org/10.3390/cancers11010070.

[6] Gasparello, J.; Papi, C.; Allegretti, M.; Giordani, E.; Carboni, F.; Zazza, S.; Pescarmona, E.; Romania, P.; Giacomini, P.; Scapoli, C.; et al. A Distinctive MicroRNA (MiRNA) Signature in the Blood of Colorectal Cancer (CRC) Patients at Surgery. *Cancers (Basel)*, **2020**, *12* (9). https://doi.org/10.3390/cancers12092410.

[7] Wan, J. C. M.; Massie, C.; Garcia-Corbacho, J.; Mouliere, F.; Brenton, J. D.; Caldas, C.; Pacey, S.; Baird, R.; Rosenfeld, N. Liquid Biopsies Come of Age: Towards Implementation of Circulating Tumour DNA. *Nat Rev Cancer*, **2017**, *17* (4), 223–238. https://doi.org/10.1038/nrc.2017.7.

[8] Li, M.; Chen, W.-D.; Papadopoulos, N.; Goodman, S. N.; Bjerregaard, N. C.; Laurberg, S.; Levin, B.; Juhl, H.; Arber, N.; Moinova, H.; et al. Sensitive Digital Quantification of DNA Methylation in Clinical Samples. *Nat Biotechnol*, **2009**, *27* (9), 858–863. https://doi.org/10.1038/nbt.1559.

[9] Radpour, R.; Barekati, Z.; Kohler, C.; Lv, Q.; Bürki, N.; Diesch, C.; Bitzer, J.; Zheng, H.; Schmid, S.; Zhong, X. Y. Hypermethylation of Tumor Suppressor Genes Involved in Critical Regulatory Pathways for Developing a Blood-Based Test in Breast Cancer. *PLoS One*, **2011**, *6* (1), e16080. https://doi.org/10.1371/journal.pone.0016080.

[10] Locke, W. J.; Guanzon, D.; Ma, C.; Liew, Y. J.; Duesing, K. R.; Fung, K. Y. C.; Ross, J. P. *DNA Methylation Cancer Biomarkers: Translation to the Clinic*; Frontiers Media S.A., 2019; Vol. 10. https://doi.org/10.3389/fgene.2019.01150.

[11] Gallardo-Gómez, M.; Moran, S.; Páez de la Cadena, M.; Martínez-Zorzano, V. S.; Rodríguez-Berrocal, F. J.; Rodríguez-Girondo, M.; Esteller, M.; Cubiella, J.; Bujanda, L.; Castells, A.; et al. A New Approach to Epigenome-Wide Discovery of Non-Invasive Methylation Biomarkers for Colorectal Cancer Screening in Circulating Cell-Free DNA Using Pooled Samples. *Clin Epigenetics*, **2018**, *10*, 53. https://doi.org/10.1186/s13148-018-0487-y.

[12] Hurd, P. J.; Nelson, C. J. Advantages of Next-Generation Sequencing versus the Microarray in Epigenetic Research. *Brief Funct Genomic Proteomic*, **2009**, *8* (3), 174–183. https://doi.org/10.1093/bfgp/elp013.

[13] Wang, Y.-M.; Trinh, M. P.; Zheng, Y.; Guo, K.; Jimenez, L. A.; Zhong, W. Analysis of Circulating Non-Coding RNAs in a Non-Invasive and Cost-Effective Manner. *Trends Analyt Chem*, **2019**, *117*, 242–262. https://doi.org/10.1016/j.trac.2019.07.001.

[14] Metcalf, G. A. D.; Shibakawa, A.; Patel, H.; Sita-Lumsden, A.; Zivi, A.; Rama, N.; Bevan, C. L.; Ladame, S. Amplification-Free Detection of Circulating MicroRNA Biomarkers from Body Fluids Based on Fluorogenic Oligonucleotide-Templated Reaction between Engineered Peptide Nucleic Acid Probes: Application to Prostate Cancer Diagnosis. *Anal Chem*, **2016**, *88* (16), 8091–8098. https://doi.org/10.1021/acs.analchem.6b01594.

[15] Shukla, N.; Yan, I. K.; Patel, T. Multiplexed Detection and Quantitation of Extracellular Vesicle RNA Expression Using NanoString. *Methods Mol Biol*, **2018**, *1740*, 177–185. https://doi.org/10.1007/978-1-4939-7652-2_14.

[16] Armstrong, D. A.; Green, B. B.; Seigne, J. D.; Schned, A. R.; Marsit, C. J. MicroRNA Molecular Profiling from Matched Tumor and Bio-Fluids in Bladder Cancer. *Mol Cancer*, **2015**, *14*, 194. https://doi.org/10.1186/s12943-015-0466-2.

[17] Ortega, F. G.; Lorente, J. A.; Garcia Puche, J. L.; Ruiz, M. P.; Sanchez-Martin, R. M.; de Miguel-Pérez, D.; Diaz-Mochon, J. J.; Serrano, M. J. MiRNA in Situ Hybridization in Circulating Tumor Cells--MishCTC. *Sci Rep*, **2015**, *5*, 9207. https://doi.org/10.1038/srep09207.

[18] Li, L.; Lu, M.; Fan, Y.; Shui, L.; Xie, S.; Sheng, R.; Si, H.; Li, Q.; Wang, Y.; Tang, B. High-Throughput and Ultra-Sensitive Single-Cell Profiling of Multiple MicroRNAs and Identification of Human Cancer. *Chem Commun (Camb)*, **2019**, *55* (70), 10404–10407. https://doi.org/10.1039/c9cc05553c.
